# Displaying and delivering viral membrane antigens via WW domain–activated extracellular vesicles

**DOI:** 10.1126/sciadv.ade2708

**Published:** 2023-01-27

**Authors:** Sengjin Choi, Zhiping Yang, Qiyu Wang, Zhi Qiao, Maoyun Sun, Joshua Wiggins, Shi-Hua Xiang, Quan Lu

**Affiliations:** ^1^ Program in Molecular and Integrative Physiological Sciences, Department of Environmental Health, Harvard T.H. Chan School of Public Health, Boston, MA 02115, USA.; ^2^ Nebraska Center for Virology, School of Veterinary Medicine and Biomedical Sciences, University of Nebraska-Lincoln, Lincoln, NE 68583, USA.

## Abstract

Membrane proteins expressed on the surface of enveloped viruses are conformational antigens readily recognized by B cells of the immune system. An effective vaccine would require the synthesis and delivery of these native conformational antigens in lipid membranes that preserve specific epitope structures. We have created an extracellular vesicle–based technology that allows viral membrane antigens to be selectively recruited onto the surface of WW domain–activated extracellular vesicles (WAEVs). Budding of WAEVs requires secretory carrier-associated membrane protein 3, which through its proline-proline-alanine-tyrosine motif interacts with WW domains to recruit fused viral membrane antigens onto WAEVs. Immunization with influenza and HIV viral membrane proteins displayed on WAEVs elicits production of virus-specific neutralizing antibodies and, in the case of influenza antigens, protects mice from the lethal viral infection. WAEVs thus represent a versatile platform for presenting and delivering membrane antigens as vaccines against influenza, HIV, and potentially many other viral pathogens.

## Teaser

Extracellular vesicles are engineered to present and deliver viral membrane antigens as vaccines.

## INTRODUCTION

Viral pathogens pose grave threats to public health as evidenced by the reoccurring flu epidemics, the HIV epidemic, and the current coronavirus disease 2019 (COVID-19) pandemic. The most effective way to stop viral infections and their ensuing ravages is vaccination (*1*, *2*). While there are a variety of ways to vaccinate, a common approach involves the use of viral proteins as antigens to stimulate the immune system to develop protection against the infection (*3*, *4*). Because membrane proteins expressed on the surface of an enveloped virus are readily recognized by B cells of the immune system that produce neutralizing antibodies, they are often the preferred antigens in vaccine development (*3*, *4*). These viral membrane proteins include the hemagglutinin (HA) and Matrix 2 (M2) ion channel proteins of influenza virus (*5*), the envelope protein of HIV (*6*, *7*), and the spike protein of severe acute respiratory syndrome coronavirus 2 (SARS-CoV-2) (*8*, *9*).

Viral membrane proteins often contain extracellular and cytosolic regions that are linked together by a transmembrane domain (TM). The TM anchors the viral protein on the host cell membrane before viral egress and then on the viral membrane after egress. While the extracellular region generally contains most antigenic epitopes, it often needs to be expressed along with the TM within the lipid membrane to assume its correct conformation for optimal epitope presentation and immune response. For example, much effort has been focused on coaxing the HIV envelope protein into a prefusion trimer conformation; yet, such effort has not been successful partly because of the relative instability of the trimer in the absence of lipid membrane (*10*). A smaller region known as the membrane-proximal external region (MPER) in the HIV envelope protein is half-embedded within the lipid bilayer and has been used to develop HIV vaccine (*10*, *11*); however, synthetic MPER peptide needs to be mixed with lipid to improve its immunogenicity (*12*). Similarly, recombinant flu M2 protein requires the lipid membrane to elicit effective antibody response (*13*, *14*). Thus, delivering these viral membrane antigens in their native conformation associated with lipid membrane remains a challenge in vaccine development.

Extracellular vesicles (EVs) are small nanoscale vesicles secreted by almost all mammalian cells (*15*, *16*). Some EVs such as arrestin domain containing 1 (ARRDC1)–mediated microvesicles (ARMMs) bud directly from the plasma membrane (*17*, *18*). Budding of ARMMs requires the recruitment of cellular proteins such as TSG101 and other endosomal sorting complexes required for transport (ESCRT) components (*17*), which are also often the host proteins used by viruses such as HIV and Ebola for their egress from the host cell (*19*–*21*). ARMMs and potentially other EVs thus can be considered endogenous viral-like particles—they bud similar to viruses from the plasma membrane, are generally in the same size range as most viruses, and are encapsulated by host cell membrane with transmembrane proteins on the surface. While EVs such as ARMMs are often considered less immunogenic (*22*, *23*), their structural and functional analogy with budding viruses suggests a possibility that viral antigens may be engineered onto the surface of these EVs to elicit immune response.

Here, we explored EVs as a way to present viral membrane antigens for vaccine development. Our work unexpectedly found a way of making EVs, whose budding is driven by interaction of WW domains with a membrane protein SCAMP3 (secretory carrier-associated membrane protein 3). Using WW domain–activated EVs (WAEVs), we presented and delivered a variety of viral membrane proteins, including two flu viral proteins and the MPER peptide of HIV. Administration of these viral membrane antigens via WAEVs elicited production of specific viral antibodies and, in the case of flu, protected mice from lethal challenges of influenza infection. Our study established WAEVs as a versatile platform for delivering membrane protein antigens as vaccines for flu, HIV, and likely other human viral pathogens.

## RESULTS

### Fusion to WW domain promotes budding of EVs

**Fig. 1.**
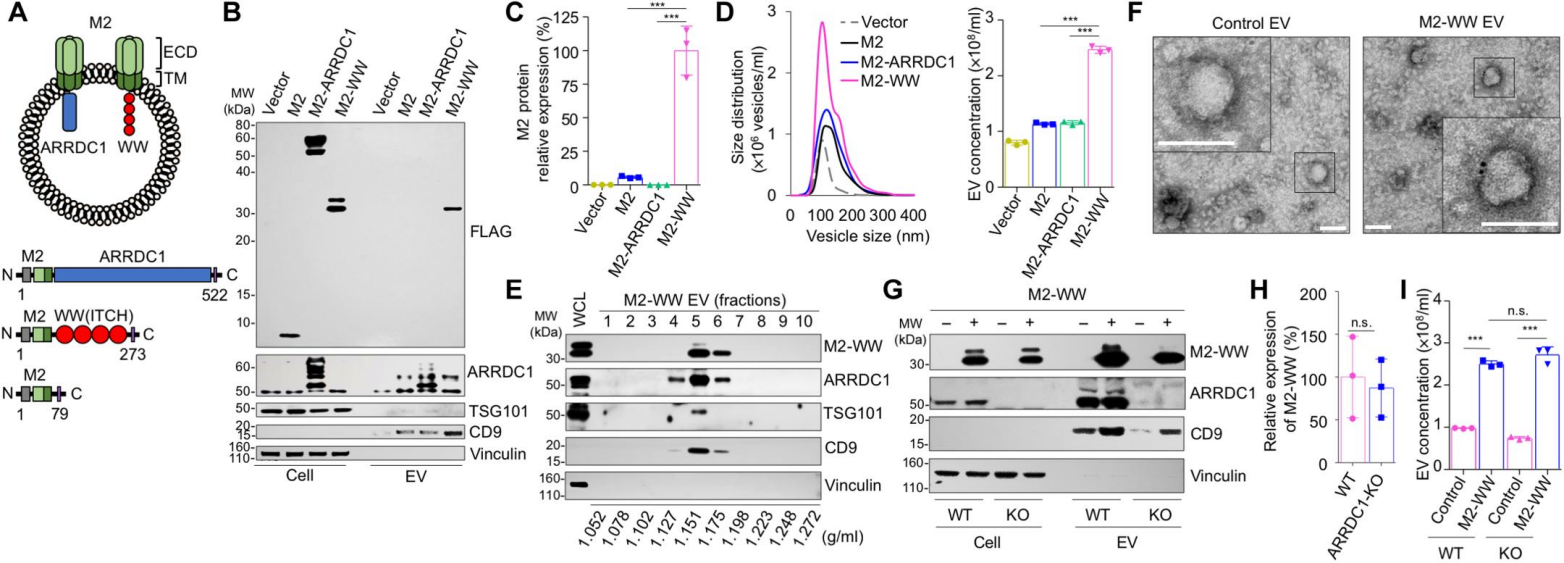
Budding of M2-WW fusion protein into EVs. (**A**) Schematic drawings of EVs with influenza viral M2 and of various constructs. (**B**) Western blotting showing budding of M2-WW fusion proteins into EVs in HEK293T cells. EVs were isolated via ultracentrifugation. Western blotting was done on the EVs along with whole-cell lysates with indicated antibodies. MW, molecular weight. (**C**) Western blotting data were analyzed via image quantification using ImageJ software. Each band intensity of EV was normalized with the band intensity of cell lysate of same amount. Western blotting was repeated three times to obtain the data for each band. ***P < 0.001. (**D**) NanoSight particle analysis of EVs. Data in the NanoSight particle analysis were obtained from triplicates for each condition. ***P < 0.001. (**E**) Fractionation of M2-WW EVs by OptiPrep-based density gradient ultracentrifugation. EVs from M2-WW–transfected HEK293T cells were isolated via ultracentrifugation and subject to OptiPrep density gradient ultracentrifugation. Ten fractions were obtained, followed by ultracentrifugation. Western blotting was done on the EV fractions along with whole-cell lysates with indicated antibodies. The density for each of the fractions is indicated. (**F**) Immunogold labeling and electron microscopy of control and M2-WW EVs. Scale bars, 100 nm. (**G** to **I**) M2-WW budding does not require ARRDC1. Control vector or M2-WW was transfected into either wild-type or *ARRDC1*–knockout (KO) HEK293T cells. EVs isolated from the cells were analyzed by both Western blotting (G) and NanoSight particle analysis (I). (H) Analysis of Western blotting via image quantification. Data in the Western blotting analysis were obtained from triplicates for each condition. Each band intensity of EV was normalized with intensity of cell respectively. n.s., not significant. (**I**) NanoSight particle analysis of EVs. Data were obtained from triplicates for each condition. n.s., not significant; ***P < 0.001.

We first tested the ability of ARMMs to recruit and load the M2 protein of influenza A virus. M2 is a transmembrane protein that forms a proton channel on the viral envelope (*24*). Because of its relatively high conservation among different flu strains, M2 is considered a potential target for the development of a universal flu vaccine (*25*, *26*). Our previous studies have shown that protein cargos can be recruited into ARMMs via either direct fusion to the ARRDC1 protein or to the WW domains that interact with the PPXY motifs of ARRDC1 (*27*). To test whether ARRDC1 or WW domains could be used to present M2 protein onto the surface of ARMMs, we made two fusion constructs: one containing the extracellular domain (ECD) and TM of the M2 protein fused to ARRDC1 and the other with M2 fused to the four WW domains from the ITCH protein (Fig. 1A). We then transfected the constructs into human embryonic kidney (HEK) 293T cells and collected both cell lysates and EVs for Western blotting. To our surprise, while well-expressed in the transfected cells, M2-ARRDC1 fusion protein was not detected in EVs, suggesting that fusion to ARRDC1 failed to recruit M2 into ARMMs (Fig. 1, B and C). NanoSight particle analysis confirmed this result as expression of M2-ARRDC1 in HEK293T cells did not result in an increase in EV production as compared to the control M2 expression alone (Fig. 1D). It is possible that direct fusion to a transmembrane protein such as M2 immobilizes ARRDC1 and, thus, inhibits its budding activity.

In contrast to the ARRDC1 fusion, M2 fusion to WW domains resulted in secretion of the M2-WW protein into EVs as detected by the Western blotting (Fig. 1, B and C). Moreover, M2-WW fusion led to a robust increase in the number of EVs produced (Fig. 1D). We further characterized M2-WW EVs using OptiPrep-based density gradient fractionation. M2-WW EVs were fractionated into 10 fractions on the density gradient. The peak of M2-WW EVs as indicated by M2 (FLAG-tagged) Western blotting occurred in the same fraction as that of ARMMs as indicated by ARRDC1 and that of exosomes (indicated by the exosomal marker CD9) (Fig. 1E), suggesting that M2-WW EVs are of similar size as ARMMs and exosomes. To confirm that M2 protein is presented on the surface of the EVs, we performed immunogold staining of unpermeabilized M2-WW EVs using an M2-specific antibody that recognizes the ECD of the protein. Electron microscopy of immunogold-stained EVs showed the presence of gold particles on the surface of M2-WW EVs but not on control EVs (Fig. 1F). Together, these results indicate that fusion to WW domains allow M2 protein to be recruited and displayed onto the surface of EVs.

### M2-WW EV budding is independent of ARRDC1

Because WW domains of the ITCH protein interact with ARRDC1 (*18*), we reasoned that overexpression of ARRDC1 may increase the recruitment of M2-WW fusion protein into EVs. We thus cotransfected M2-WW with either ARRDC1–green fluorescent protein (GFP) or control GFP into HEK293T cells and collected EVs for Western blotting and NanoSight analysis. While, as expected, ARRDC1-GFP robustly budded into EVs and increased EV production, to our surprise, it did not markedly increase the amount of M2-WW protein in the EVs (fig. S1), suggesting that M2-WW did not bud into ARMMs. To further confirm this, we tested M2-WW budding in *ARRDC1*-knockout (*ARRDC1*-KO) HEK293T cells in which *ARRDC1* was knocked out via CRISPR-Cas9. As shown in Fig. 1 (G and H), M2-WW fusion protein budded out into EVs in the *ARRDC1*-KO cells as efficiently as in the wild-type cells. NanoSight analysis showed that although there was a reduction in the overall EV population in *ARRDC1*-KO cells, M2-WW produced similar EV production in both *ARRDC1*-KO and wild-type cells (Fig. 1I). These results clearly indicate that ARRDC1 is not required for the budding of M2-WW into EVs. Because budding of M2-WW EVs is primarily driven by the fusion to WW domains, we named these EVs as WAEVs.

### WAEV budding is mediated by SCAMP3

**Fig. 2.**
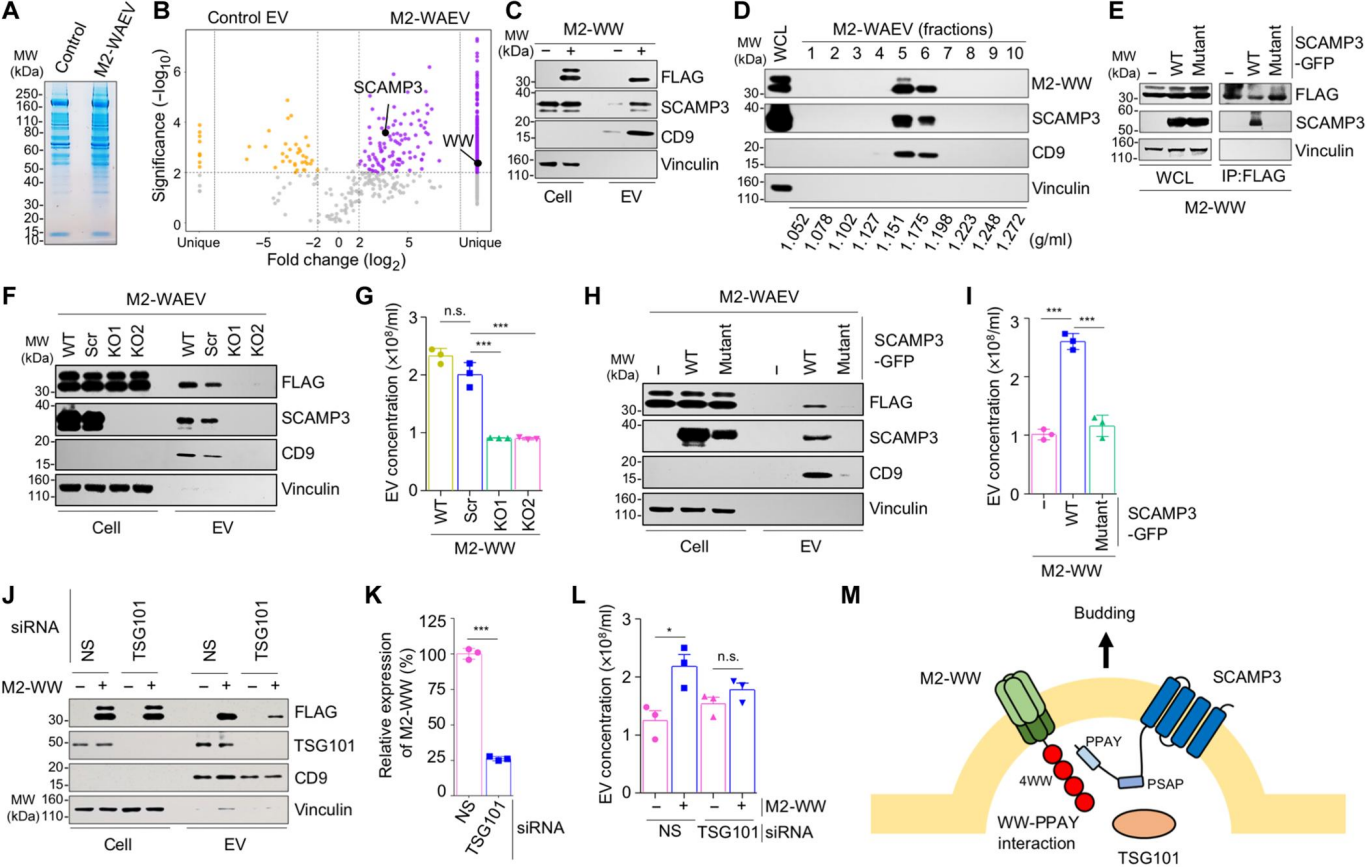
Budding of WAEVs is mediated by SCAMP3. (**A**) SDS-PAGE gel with Coomassie blue staining of proteins from control and M2-WAEVs. About 6 × 10^9^ of EVs were loaded. (**B**) Volcano plot of proteins in WAEVs. Up-regulated are marked as orange; down-regulated are marked as purple. (**C**) Western blotting showing increased presence of SCAMP3 in M2-WAEVs. (**D**) SCAMP3 cosegregates with M2-WW in fractionated EVs. M2-WAEVs were isolated via ultracentrifugation and subject to OptiPrep density gradient ultracentrifugation. (**E**) SCAMP3 coimmunoprecipitates with M2-WW. HEK293T cells were transfected with M2-WW (FLAG-tagged) along with either wild-type or mutant SCAMP3 (GFP-tagged). Immunoprecipitation (IP) was done using an anti-FLAG antibody conjugated beads. (**F** and **G**) Effect of CRISPR-based KO of *SCAMP3* on M2-WAEV budding. HEK293T cells stably transduced with lentiviruses containing scrambled gRNA or *SCAMP3*-targeting gRNAs were transfected with M2-WW. EVs were isolated from the cells and subject to Western blotting (F) or NanoSight particle analysis (G). Data were obtained from triplicates for each condition. n.s., not significant; ***P < 0.001. (**H** and **I**) Effect of reexpression of wild-type or mutant *SCAMP3* in *SCAMP3*-KO cells on M2-WAEV budding. HEK293T cells with *SCAMP3*-KO (KO1) transfected with wild-type or mutant *SCAMP3*. Both *SCAMP3* constructs contain silent mutations in the gRNA targeting region so are resistant to the CRISPR targeting. EVs were isolated from the cells and subject to Western blotting (H) or NanoSight particle analysis (I). Data in the vesicle number analysis were obtained from triplicates for each condition. ***P < 0.001. (**J**) Effect of siRNA-based knockdown of *TSG101* on M2-WAEV budding. EVs were isolated from control or *TSG101* knockdown cells and analyzed by Western blotting. (**K**) Quantification of Western blotting data in (J). ***P < 0.001. (**L**) NanoSight particle analysis of EVs. Data were obtained from triplicates for each condition. *P < 0.05. (**M**) A model of WAEV budding.

To further understand the nature of WAEVs and its budding, we next performed proteomics to identify the protein components of the vesicles. Using the OptiPrep-based density gradient ultracentrifugation method, we fractionated the EVs from M2-WW or the empty vector–transfected HEK293T cells. Peak fractions containing M2-WAEVs or the control EVs (three replicates each) were collected, and proteins in the EVs were resolved by SDS–polyacrylamide gel electrophoresis (PAGE) (Fig. 2A) and subjected to liquid chromatography–tandem mass spectrometry (LC-MS/MS). Comparison of the MS results from M2-WAEVs and control EVs identified 362 proteins that occur in all three WAEV replicates but not in any of the control EV samples (Fig. 2B and table S1). As expected, ITCH-WW peptides were only found in WAEVs. We also identified over 164 proteins, whose presence is increased (>2-fold) in the WAEVs (Fig. 2B and table S2). Further comparison of WAEV proteomic dataset with known exosomal protein database showed that, while most proteins in control EVs and WAEVs were found in exosomes (fig. S2), 12 proteins enriched in WAEVs were not previously found in exosomes (fig. S2C and table S3).

We reasoned that one or more of the enriched or specific WAEV proteins may mediate the biogenesis of the vesicles. Because WAEV budding is driven by WW domains, a potential candidate protein that can carry out WAEV budding function would likely contain PPXY motif(s) to allow for interaction with WW domains. Furthermore, the protein ideally would localize to or associate with cell membrane, where WAEV budding occurs. Analysis of the WAEV proteomic dataset identified SCAMP3 as such a potential candidate. SCAMP3, an integral membrane protein with four TMs, contains a proline-proline-alanine-tyrosine (PPAY) motif at its N-terminal cytosolic segment, which has been previously shown to interact with WW domains (*28*). Consistent with the MS result, Western blotting showed an increased amount of SCAMP3 in M2-WAEVs as compared to the control EVs (Fig. 2C). Density gradient fractionation of M2-WAEVs showed that SCAMP3 protein cosegregated well with M2-WW (Fig. 2D). The peak WAEV signal matched with that of CD9 (Fig. 2D), suggesting that SCAMP3-containing WAEVs are of similar size as classical CD9-positive exosomes. To confirm the interaction between SCAMP3 and the M2-WW fusion protein, we performed coimmunoprecipitation (co-IP) experiment. We cotransfected cells with FLAG-tagged M2-WW along with wild-type SCAMP3 or a mutant SCAMP3 that has its PPAY motif mutated to PPAA. We then used anti-FLAG antibody to pull down M2-WW protein and its associated proteins. As shown in Fig. 2E, while both wild-type and mutant SCAMP3 proteins were well expressed in cells, we detected only wild-type but not the PPAA mutant SCAMP3 protein in co-IP lysates. This result indicates that M2-WW interacts with SCAMP3 and that this interaction requires the PPAY motif in SCAMP3.

We next determined whether SCAMP3 is required for M2-WAEV budding. CRISPR targeting with two of the guide RNAs (gRNAs) led to significant reduction in SCAMP3 protein expression and consequently decreased the amount of M2-WW protein in EVs (fig. S3, A and B). Consistent with this result, NanoSight analysis showed significantly reduced number of EVs in the *SCMAP3*-knockdown cells as compared to wild-type or scramble control cells (fig. S3C). To further confirm the effect of *SCAMP3*-KO on WAEV budding, we isolated individual cell clones with clean *SCAMP3*-KO (fig. S4). In two of the sequence-confirmed, independent *SCAMP3*-KO cell clones (#8 and #13 in fig. S4B), M2-WAEV budding was almost completely abolished as evidenced by the absence of M2-WW protein in EVs (Fig. 2F) and the significant reduction of EV amount as compared to the control cells (Fig. 2G). To further establish the role of SCAMP3 in WAEV budding, we reexpressed wild-type SCAMP3, which was made resistant to CRISPR gRNA with silent mutations, in a complete *SCAMP3*-KO single clone. This reexpression increased M2-WW protein in WAEVs (Fig. 2H) and restored M2-WAEV budding (Fig. 2I). In contrast, reexpression of the PPAA SCAMP3 mutant that does not interact with the WW domains failed to increase M2-WW in EVs or to restore M2-WAEV budding (Fig. 2, H and I). These data demonstrate the essential role of SCAMP3 in WAEV budding.

A previous study has shown that SCAMP3 interacts, through its PSAP motif, with TSG101 (*28*), the ESCRT-I complex protein required for budding of ARMMs and multivesicular body (MVB) formation in late endosomes. We next tested whether TSG101 is required for WAEV budding. As shown in Fig. 2J and quantified in Fig. 2K, small interfering RNA (siRNA)–mediated knockdown of TSG101 significantly reduced the amount of M2-WW in EVs. Consistent with this result, expression of M2-WW failed to increase EV production in *TSG101*-knockdown cells (Fig. 2L). Together, our data strongly support a model in which SCAMP3 mediates M2-WAEV budding via its interactions with the WW domains and TSG101 (Fig. 2M).

### Immunization with M2-WAEVs protected mice from lethal flu virus challenge

**Fig. 3.**
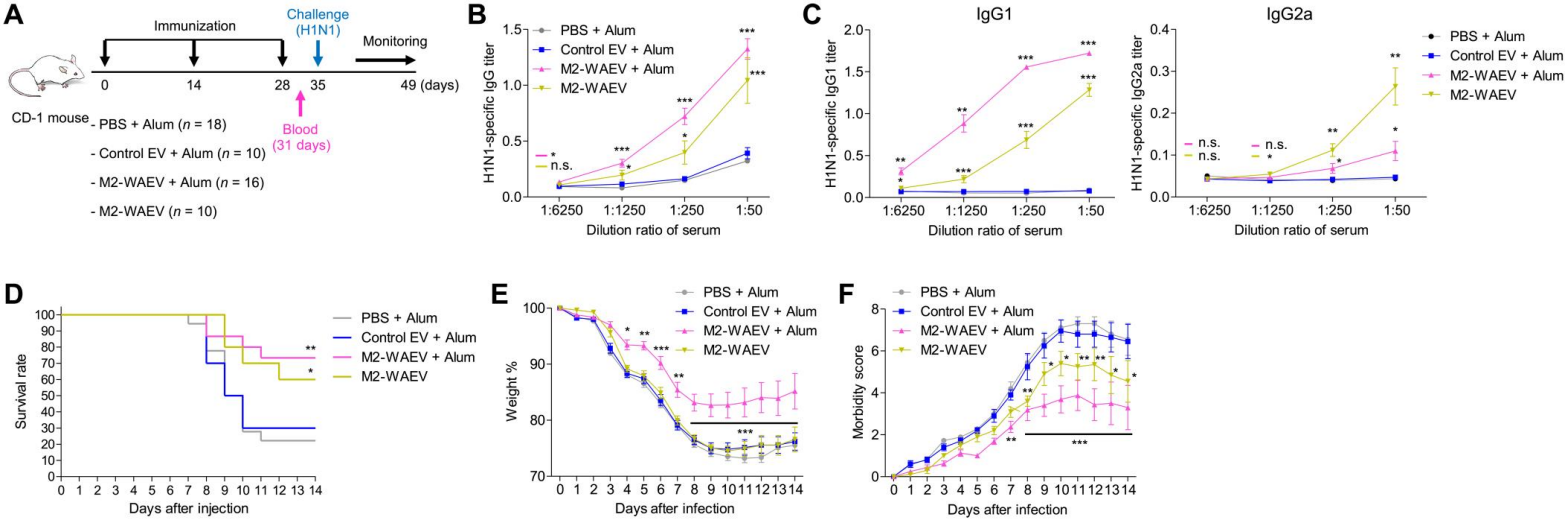
M2-WAEV immunization elicits antibody production and protects mice against H1N1 viral infection. (**A**) Three-shot immunization protocol. CD-1 mice (Charles River Laboratories) were immunized via intraperitoneal injection with one of the following: PBS with Alum as an adjuvant, control EVs (no M2) with Alum, M2-WAEVs with Alum, or M2-WAEVs without Alum. Sera were collected from mice 3 days after final immunization. One week after the final immunization, all mice were subjected to H1N1 influenza viral infection [strain A/Puerto Rico/8/1934/H1N1 at 800 plaque-forming units (PFU); given intranasally] and followed by morbidity and mortality measurement for 2 weeks. (**B**) Levels of H1N1-reactive IgG in serum. Inactivated whole influenza virus (A/Puerto Rico/8/1934/H1N1) was used to coat the 96-well plate. Antibody response was measured using indirect ELISA using serially diluted serum (1:6250, 1:1250, 1:250, and 1:50). (**C**) Levels of H1N1-reactive IgG subtypes (IgG1 and IgG2a) in serum. (**D**) Survival rate of immunized mice after influenza virus infection. Mortality was monitored every day for 2 weeks after influenza virus infection. (**E**) Weight measurement in immunized mice after influenza virus infection. (**F**) Morbidity in immunized mice after influenza virus infection. Morbidity was scored on 0 to 8 scale (higher number for more morbid). *P < 0.05, **P < 0.01, and ***P < 0.001.

We next tested the effect of M2-WAEVs as a vaccine using a mouse model of flu infection. CD-1 mice susceptible to influenza viral infection were immunized (via intraperitoneal injection) with M2-WAEVs three times during a 4-week period in the presence or absence of adjuvant aluminum hydroxide (Alum) (Fig. 3A). Controls included mice injected with phosphate-buffered saline (PBS) with Alum or empty EVs that did not contain M2. The dose of WAEVs we used was 5 × 10^9^ per mouse per injection. This corresponds to about 200 ng of M2 peptide on WAEVs (fig. S5). During the immunization process, we did not observe any toxic effects in immunized mice as evidenced by no change in body temperature, no change in weight, and no significant change in serum inflammatory cytokines (fig. S6, B and C). We also did not observe any tissue toxicity in the liver or kidney (fig. S6, G and H). Three days after final immunization, mouse sera were collected and checked for antibody production by enzyme-linked immunosorbent assay (ELISA). A week after the final immunization, all mice were intranasally infected with H1N1 influenza virus (mouse-adapted, Puerto Rico 8 strain). Infected mice were monitored for morbidity and mortality for 2 weeks. The ELISA result showed that sera from mice injected with M2-WAEVs had immunoglobulin G (IgG) antibodies that bind to influenza A virus (Fig. 3B). Although the antibody titer was highest in the Alum group, the M2-WAEV group without Alum also elicited antibody response that is significantly higher than the background level observed in the two control groups (PBS or control EVs) (Fig. 3B). Although both IgG1 and IgG2a subtypes were induced, M2-WAEV without Alum induced more IgG2a subtype (Fig. 3C). Within 2 weeks of flu infection, >70% mice in the control groups (PBS or control EVs with Alum) died, whereas ~70% mice immunized with M2-WAEVs (with Alum) survived (Fig. 3D). Mice that received M2-WAEVs without Alum also showed a significantly improved survival rate (60%) (Fig. 3D). Consistent with the mortality data, mice that received M2-WAEVs with Alum showed significantly less body weight loss (Fig. 3E) and lower morbidity (Fig. 3F) than the control groups. These data indicate that immunization of mice with M2-WAEVs elicited production of antibodies and protected the animals from flu infection.

We next tested whether a single-dose administration of M2-WAEVs offers protection against the flu infection (fig. S7A). CD-1 mice were administered via intraperitoneal injection with M2-WAEVs, control EVs, or PBS. All groups contained the adjuvant Alum. Thirty-one days after the immunization, mouse sera were collected. Five weeks (same timeline as the three-dose regimen) after the immunization, all mice were intranasally infected with the PR8 H1N1 influenza virus and followed up for two additional weeks. We detected H1N1-binding antibodies in M2-WAEV–immunized mice but not in the two control groups (fig. S7B), although the level of antibodies was about threefold lower than that in the three-dose regimen (Fig. 3B). Similarly, mice immunized with a single dose of M2-WAEVs exhibited better survival rate, less weight loss, and lower morbidity against the flu infection as compared to the two control groups (fig. S7, C to E), but the effect of protection (i.e., the difference between the M2-WAEV group and the control groups) is lower than that of three-dose administration. Together, these data showed that different regimens of M2-WAEVs immunization all elicited antibody production and reduced the lethality of influenza viral infection.

### Immunization with WAEVs containing the flu HA stalk region also protects mice from flu infection

**Fig. 4.**
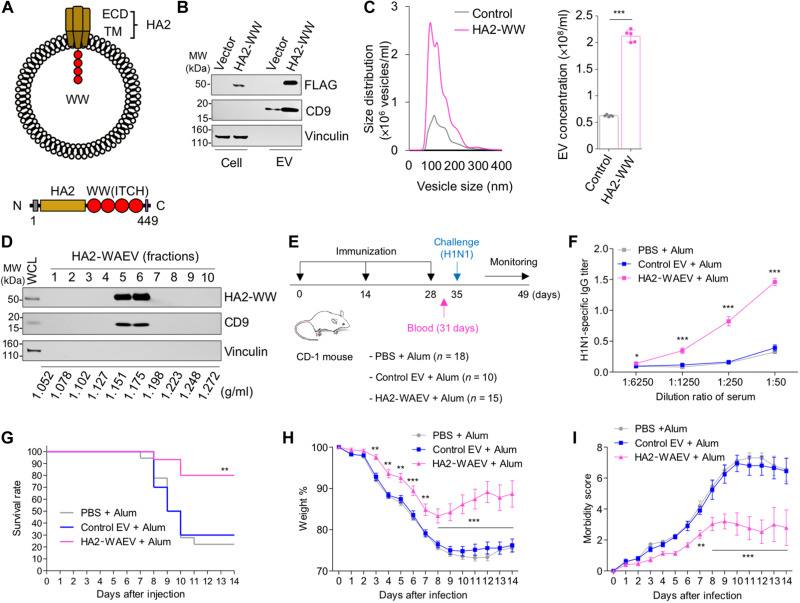
HA2-WAEV immunization elicits antibody production and protects mice against H1N1 viral infection. (**A**) Schematic drawings of HA2-WAEVs and of HA2-WW construct. (**B**) Budding of HA2-WW fusion protein into EVs in HEK293T cells. Control or HA2-WW constructs were transfected into HEK293T cells. Forty-eight hours after transfection, EVs were isolated via ultracentrifugation and analyzed by Western blotting along with whole-cell lysates with indicated antibodies. (**C**) NanoSight particle analysis of EVs collected from HEK293T cells transfected with vector control or HA2-WW construct. The *y* axis represents particle concentration at each nanometer interval. (**D**) Fractionation of HA2-WAEVs by OptiPrep-based density gradient ultracentrifugation. EVs from M2-WW–transfected HEK293T cells were isolated via ultracentrifugation and subject to OptiPrep density gradient ultracentrifugation. Ten fractions were obtained, followed by ultracentrifugation. Western blotting was done on the EV fractions along with whole-cell lysates with indicated antibodies. The density for each of the fractions is indicated. (**E**) HA-2WAEV mouse immunization procedure. CD-1 mice (Charles River Laboratories) were immunized via intraperitoneal injection with one of the following: PBS, control EVs, and HA2-WAEVs (all with Alum). Sera were collected from mice 3 days after the final immunization. One week after the final immunization, all mice were subjected to H1N1 influenza viral infection (strain A/Puerto Rico/8/1934/H1N1 at 800 PFU; given intranasally) and followed by morbidity and mortality measurement for 2 weeks. (**F**) Levels of H1N1-reactive IgG in serum. Antibody response was measured using indirect anti-H1N1 ELISA using a serially diluted serum. (**G**) Survival rate of immunized mice after influenza virus infection. Mortality was monitored every day for 2 weeks after influenza virus infection. (**H**) Weight measurement in immunized mice after influenza virus infection. (**I**) Morbidity in immunized mice after influenza virus infection. Morbidity was scored on a 0 to 8 scale (higher number for more morbid). *P < 0.05, **P < 0.01, and ***P < 0.001.

We next tested whether WAEVs could be used for presentation of another membrane protein of the influenza virus: the HA. The stalk region of HA known as HA2 is relatively less variable than the head region (HA1) of the protein and is thus also considered as a potential target of vaccine development (*29*, *30*). We made a fusion construct in which WW domains of ITCH protein was fused to HA2 and the TM of the HA protein (Fig. 4A). Transfection and expression of this construct in HEK293T cells led to the budding of HA2-WW fusion protein into EVs (Fig. 4B) and significantly increased vesicle production (Fig. 4C). When fractionated by density gradient ultracentrifugation, HA2-WAEVs had peak fractions at density of 1.151 and 1.175 g/ml, which are similar to M2-WAEVs (Fig. 4D).

We next determined whether HA2-WAEVs could be used as a vaccine to protect mice from flu infection. CD-1 mice were immunized (via intraperitoneal injection) three times over 4 weeks with HA2-WAEVs, PBS, or empty EVs that did not contain HA2 (Fig. 4E). Sera from mice injected with HA2-WAEVs contained antibodies that bind to influenza A virus (Fig. 4F). A week after the final immunization, all mice were intranasally infected with H1N1 influenza virus and monitored for morbidity and mortality for 2 weeks (Fig. 4E). While >70% mice in the control groups died, ~80% mice immunized with the HA2-WAEVs survived the viral infection (Fig. 4G). Consistent with the mortality data, mice that received HA2-WAEVs showed significantly less body weight loss (Fig. 4H) and lower morbidity (Fig. 4I) than the control groups. Together, these data indicated that, similar to M2-WAEVs, HA2-WAEV immunization elicited viral antibody production and protected mice against the lethality of influenza viral infection.

### WAEVs containing an HIV envelope peptide produce anti-HIV neutralizing antibodies

**Fig. 5.**
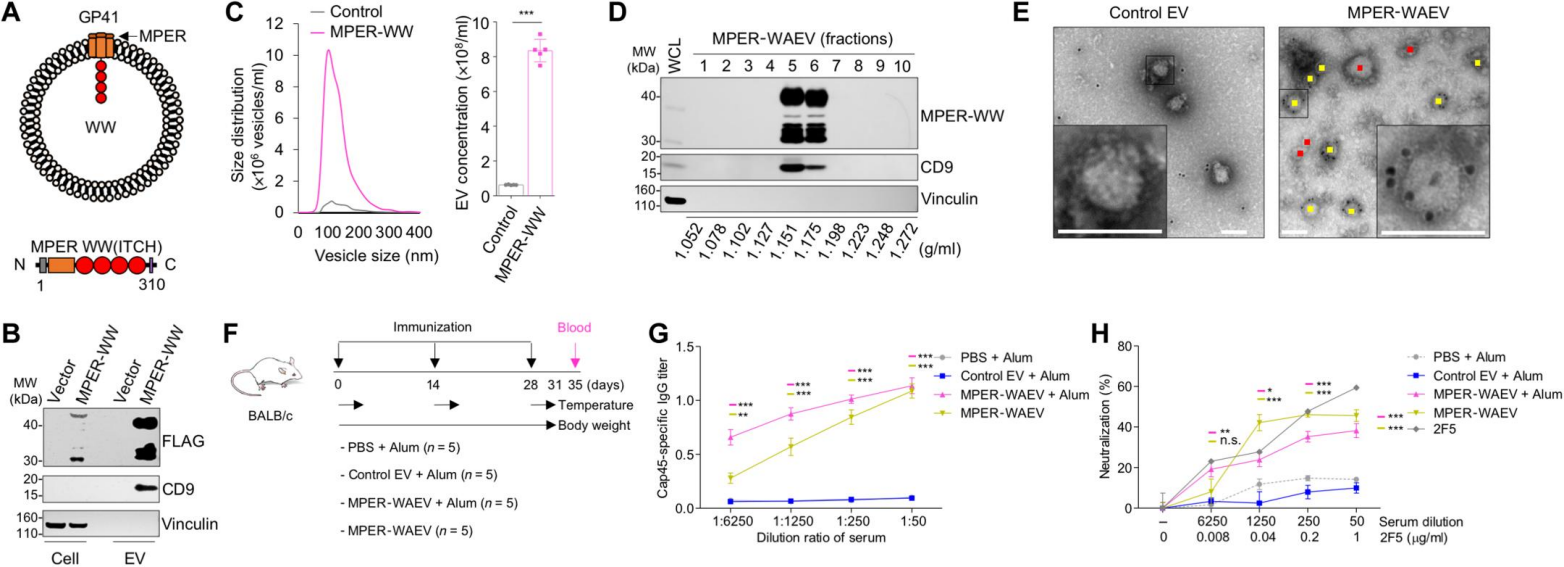
MPER-WAEVs immunization elicits production of HIV-neutralizing antibodies. (**A**) Schematic drawings of MPER-WAEV and of the MPER-WW fusion construct. (**B**) Budding of MPER-WW fusion protein into EVs in HEK293T cells. Control or MPER-WW constructs were transfected into HEK293T cells. Forty-eight hours after transfection, EVs were isolated via ultracentrifugation and analyzed by Western blotting. (**C**) NanoSight particle analysis of EVs collected from HEK293T cells transfected with vector control or MPER-WW construct. (**D**) Fractionation of MPER-WAEVs by OptiPrep-based density gradient ultracentrifugation. EVs from MPER-WW–transfected HEK293T cells were isolated via ultracentrifugation, subject to OptiPrep density gradient ultracentrifugation, and analyzed by Western blotting. (**E**) Electron microscopy images of immunogold staining of EVs. Control EVs or MPER-WAEVs were purified via sucrose density gradient ultracentrifugation and then incubated with anti-MPER 2F5 primary antibody, followed by gold particle–conjugated secondary antibody. Vesicles were imaged by transmission electron microscope. Scale bars, 100 nm. MPER-positive and MPER-negative EVs are marked with yellow and red dots, respectively. (**F**) MPER-WAEV immunization protocol in mice. (**G**) Levels of HIV-reactive IgG in serum. ELISA was done with diluted sera samples on 96-well plates that were coated with HIV pseudo-virus (Cap45) and blocked with 5% bovine serum albumin in PBS with Tween-20 (PBST). Each sera sample was measured in duplicates. (**H**) Viral neutralization assay of mouse serum. HIV pseudo-virus (YU2) was mixed with mouse serum (at indicated dilution) or purified recombinant 2F5 antibody (positive control) and then added to TZM-bl cells. One day after infection, cells were washed with PBS, and fresh medium was added to the cells. Three days after infection, the supernatant was removed; the cells were washed with PBS, lysed, and measured for luciferase activity. *P < 0.05, **P < 0.01, and ***P < 0.001; n.s., *P* > 0.05.

We next tested whether WAEVs can be used to present membrane protein antigens of other viruses. The MPER peptide is a relatively invariant region of the HIV envelope protein gp41 and contains epitopes targeted by multiple broad neutralizing antibodies (bNAbs) (*31*, *32*). As a result, MPER is considered an important target in HIV vaccine development (*33*, *34*). However, MPER in its native state is half-embedded in the lipid bilayer, and perhaps as a result, synthetic MPER peptide alone without membrane lipids does not elicit bNAb production (*12*). We thus tested whether MPER peptide can be displayed on the surface of WAEVs to improve its ability to induce the production of neutralizing antibodies. We made a fusion construct in which the WW domains of ITCH are fused to MPER along with the TM of the HIV envelope protein gp41 (Fig. 5A). Transfection and expression of the MPER-WW fusion construct in HEK293T cells led to the budding of MPER-WW fusion protein into EVs (Fig. 5B) and markedly increased vesicle production (Fig. 5C). MPER-WW EVs were fractionated using density gradient ultracentrifugation, and the peak fractions of MPER-WAEVs were at a density of 1.151 and 1.175 g/ml, which are similar to both M2-WAEVs and HA2-WAEVs (Fig. 5D). To determine whether the MPER peptide was displayed on the surface of WAEVs, MPER-WAEVs were first purified using the density gradient ultracentrifugation; immunogold labeling of the vesicles was then performed using an anti-HIV bNAb 2F5. Electron microscopy showed immunogold particles on the surface of the majority (8 of 12) of MPER-WAEVs but not on any of the control EVs that do not contain MPER (Fig. 5E). This result indicates that MPER was not only on the WAEVs but was also presented in the correct conformation that allows for the binding by the bNAb.

We next tested whether immunization with MPER-WAEVs induces the production of anti-HIV bNAb in vivo. BALB/c mice (4 weeks old) were immunized three times (days 0, 14, and 28) with MPER-WAEVs either with or without Alum (Fig. 5F). Controls included mice injected with PBS with Alum or empty EVs that do not contain MPER. Similar to M2-WAEV immunization, we did not observe any toxic effects in MPER-WAEV–immunized mice, as evidenced by no change in body temperature, weight, or serum inflammatory cytokines (fig. S5). A week after final immunization, mouse sera were collected and checked for antibody production by an HIV ELISA. As shown in Fig. 5G, ELISA showed that serum from MPER-WAEV–immunized animals contained antibodies that bind to HIV pseudo-virus (Cap45)–coated plate. The level of antibodies in the Alum MPER-WAEV group was higher than that in the no Alum group. These results indicate that MPER presented on WAEVs is able to induce production of antibodies that bind HIV.

We then tested whether the antibodies in the serum from MPER-WAEV–immunized mice can neutralize HIV infection. TZM-bl cells express HIV receptors CD4 and CCR5 and can be infected by pseudo-typed HIV (YU2) viruses. YU2 viruses were mixed with control or the serum samples from immunized mice (at different dilutions) and then used to infect TZM-bl cells. The bNAb 2F5 antibody was used as a positive control. Three days after infection, the cells were lysed and measured for luciferase activity. As expected, the recombinant bNAb 2F5 antibody was able to reduce HIV viral infection substantially at as low as 8 ng/ml dose (Fig. 5H). The serum from MPER-WAEV–immunized animals was able to reduce YU2 infection of TZM-bl cells in a dose (dilution fold)–dependent manner (Fig. 5H). The neutralizing effect of the MPER-WAEV serum at 1:1250 dilution is comparable to that of recombinant bNAb 2F5 antibody (40 ng/ml) (Fig. 5H). This result indicates that sera from mice immunized with MPER-WAEVs contain antibodies that neutralize HIV and can potentially prevent infection.

## DISCUSSION

In this study, we have developed an EV-based method that can present and deliver viral surface protein antigens within the environment of lipid membrane. Through fusion to WW domains, we were able to recruit and package viral membrane proteins or peptides onto the surface of WAEVs. Membrane proteins on WAEVs are likely in their natural confirmation as they can be recognized by appropriate antibodies, including neutralizing antibodies. In all cases, we showed that these viral membrane antigens containing WAEVs induce robust production of antibodies that specifically bind to viruses. In the case of the flu virus, we showed that immunization of viral antigens on WAEVs significantly reduced the lethality of the viral infection in mice. These results demonstrated the utility of WAEVs in presenting and delivering a variety of viral membrane proteins in their native membrane-associated conformation and established WAEVs as a highly adaptable platform for developing vaccines against viral pathogens.

While we initiated the studies intended to deliver viral membrane proteins using existing, known EVs such as ARMMs, our work unexpectedly found a relatively simple way of increasing the production of EVs through the expression of transmembrane proteins fused to WW domains. The resulting EVs, termed WAEVs, are distinct from ARMMs as ARRDC1 is neither enriched in nor required for WAEV budding. Numerous proteins identified in WAEVs are not previously known to be associated with ARMMs or classical exosomes. Proteomic analysis of WAEVs followed by CRISPR functional KO studies identified SCAMP3 as an important mediator of WAEV budding. Our data clearly showed that SCAMP3 mediates WAEV budding by interacting with the WW domains via its PPAY motif. SCAMP3 regulates the formation of MVB (*35*) and, furthermore, contains a PSAP motif that interacts with TSG101 (*28*), the ESCRT-I complex protein required for budding of ARMMs and, as we showed in this study, WAEVs. Thus, despite the differences between ARMMs and WAEVs, they share the same critical proline-rich motifs (PPXY and PSAP) that are required for vesicle budding. Our work supports a working model (Fig. 2M), in which the WW domains fused to viral membrane proteins interact with the PPAY motif of SCAMP3, which subsequently recruits TSG101 via its PSAP motif to the cell membrane to drive the budding of WAEVs.

Although our data clearly showed that SCAMP3 is required for the production of WAEVs, it is not the first time that SCAMP3 was implicated in EVs. A previous study showed that SCAMP3 is present on the surface of EVs collected from human mast cells (*36*). In that study, SCAMP3 was found to exist in a reversed topology; it was not clear at all whether SCAMP3 is just a bystander or is functionally involved in the biogenesis of certain endogenous population of EVs. Nevertheless, it remains a possibility that SCAMP3 recruitment by WW fusion proteins during WAEVs production may perturb the potential function of SCAMP3 in endogenous EV formation. Similarly, SCAMP3 is known to regulate trafficking to the plasma membrane as well as the degradation and recycling of epidermal growth factor receptor (EGFR) (*28*). It is possible that recruitment and incorporation of SCAMP3 into WAEVs may interfere with its physiological role in regulating membrane trafficking and EGFR signaling. Future studies are needed to examine the impact of WAEV budding on endogenous EV formation and receptor membrane trafficking.

WAEVs have several potential advantages over existing platforms of vaccination. Comparing to recombinant protein-based vaccination, which usually requires substantial and challenging protein engineering to keep the antigens in their native conformation (*3*, *37*), WAEVs bypass this obstacle as viral membrane proteins are expressed on cell membrane and loaded naturally onto the membrane of WAEVs during vesicle budding in production cells. WAEVs are of nonviral origin and, thus, are unlikely to be affected by existing or induced neutralizing antibodies that hamper adeno-associated virus and other viral-based vaccination platforms (*38*, *39*). WAEVs also may overcome some of the challenges existed for the state-of-the-art mRNA platform, which was successfully used to produce highly effective SARS-CoV-2 vaccines (*40*, *41*). For example, WAEVs are generally very stable and can be stored at 4°C, could address the relatively labile nature of mRNAs, and thus reduces the cost in production, transport, and final delivery of vaccines in resource-poor settings. Another important advantage of the WAEV-based vaccine platform is the dispensability of adjuvants, as we showed that WAEVs without the adjuvant Alum induced production of antibodies and provided protection against viral infection. Thus, WAEVs may avoid the use of adjuvants, further reducing the cost and potential side effects of vaccines (*42*, *43*).

Our current study provided a preclinical proof-of-concept demonstration for using WAEVs to deliver vaccine antigens for flu and HIV viruses. Future studies and further developments are needed to bring these vaccines into clinics. For flu viruses, a critical goal is to develop a universal vaccine that works for most flu variants and clades (*44*). M2 and HA2 are, in general, less variable regions of the flu virus although the conservation is not very broad. Thus, our M2/HA2-WAEVs could prevent viral infection of other strains that share conserved M2/HA2 epitopes, but to develop truly universal flu vaccines, selection and testing of better antigens such as multivalent design that covers more flu strains and clades are likely needed. In addition, WAEV-based flu vaccines need to be tested in relevant models such as ferrets that better mimic human infections (*45*). For HIV, MPER-WAEVs need to be tested in a relevant in vivo mouse model with humanized immune system (*46*) or the SHIV (simian HIV) model in nonhuman primates (*47*). Furthermore, for WAEV vaccines to enter human studies, the vesicles likely need to be produced in more appropriate Good Manufacturing Practice (GMP)-compliant cells such as the Expi293 cells and purified via antibody-based affinity chromatography to remove other EVs and contaminants.

While this study was focused on membrane antigens of flu and HIV viruses, the WAEV system can be used to display and deliver membrane antigens of other enveloped viruses such as SARS-CoV-2. SARS-CoV-2 has on its surface multiple membrane proteins including the spike, envelope, and membrane proteins. WAEVs engineered with these proteins on surface could lead to the production of neutralizing antibodies and protection against COVID-19 infections. WAEVs may also be used more broadly to present nonviral membrane proteins. We envision an equally effective presentation and delivery of cellular membrane receptors for production of therapeutic antibodies and membrane autoantigens for inducing immune tolerance. Further exploring WAEVs in presenting and delivering an expanding repertoire of viral and nonviral membrane protein antigens will help develop WAEVs into a highly versatile and potentially superior platform for vaccine development and beyond.

## MATERIALS AND METHODS

### Plasmid constructs

ARRDC1-GFP expression construct was prepared previously (*17*). M2-WW fusion construct was made by cloning DNA sequences corresponding to the 1 to 48 amino acid of N-terminal M2 (UniProt, P06821) fused to the four WW domains of ITCH (amino acids 327 to 511 of full-length ITCH; UniProt, Q96J02). HA2-WW fusion construct was made by cloning DNA sequences corresponding to the 344 to 565 amino acid of C-terminal HA (UniProt, P03452) fused to the ITCH WW domains. MPER-WW fusion construct was made by cloning DNA sequences corresponding to the 619 to 702 amino acid of C-terminal envelope glycoprotein gp160 (UniProt, Q75760) fused to ITCH WW domains. Signal peptide (METDTLLLWVLLLWVPGSTGD) was used for M2-WW, HA2-WW, and MPER-WW to enhance membrane presentation of the fusion proteins. M2-ARRDC1 fusion construct was made by closing DNA sequences corresponding to 1 to 48 amino acid of N-terminal M2 fused with full-length ARRDC1 (UniProt, Q8N5I2). All fusion constructs were cloned into the pcDNA3.1-c-DYK (+) vector.

### Cell culture and transfection

HEK293T cells were cultured in Dulbecco’s modified Eagle’s medium (DMEM) (Gibco) supplemented with 10% fetal bovine serum (FBS) (Gibco, 10082) and antibiotics (Gibco, 10378). HEK293T cell were grown at 37°C in 5% CO_2_ cell incubator. Transfections of plasmid on HEK293T cells were done using the TurboFect reagent (Thermo Fisher Scientific, R0531). *ARRDC1*-KO HEK293T cells were described previously (*18*) and were cultured similarly to HEK293T cells.

### CRISPR-Cas9–based gene KO

CRISPR constructs were designed and made in pLenti-CRPSIR system by GenScript (Piscataway, NJ) to target exon 4 (gRNA1 sequence: GCTGCAGCTCTCGCTCCCTT) and exon 3 (gRNA2: TGTGGGGCTGAGCTTTCTCG) of human *SCAMP3* gene. Lentiviruses produced from the CRISPR gRNA constructs were used to transduce HEK293T cells. After lentiviral transduction, stable clones were selected using puromycin selection and pooled. Individual single-cell clones were isolated by flow cytometry. KO of *SCAMP3* in mixed cell population or single clones was confirmed by Western blotting and genomic polymerase chain reaction, followed by direct DNA sequencing.

### siRNA-based knockdown

All siRNAs were made by Dharmacon Inc. (Lafayette, CO). SMARTpool siRNA targeting *TSG101* (L-003549) and nontargeting pool siRNA (D001810) were transfected into HEK293T cell using the DharmaFECT 1 Transfection Reagent (T-2001). Knockdown of TSG101 was confirmed by Western blotting.

### EV production, isolation, and analysis

Twenty-four hours after transfection, the cell culture medium was replaced with DMEM that contains EV-depleted FBS (Gibco). Seventy-two hours after transfection, cell culture supernatants were precleared via two consecutive rounds of centrifugation (500*g* for 10 min and 2000*g* for 10 min) and filtered using a 0.2-μm syringe filter (Pall Acrodisc, catalog no. 4562). EVs in the supernatants were then pelleted by ultracentrifugation using SW32 Ti rotor at 100,000*g* for 2 hours. EV pellet was resuspended in filtered PBS and used immediately or stored at 4°C. EVs were analyzed and quantified by the NanoSight NS300 instrument with NTA software (Malvern Panalytical).

### Density gradient fractionation of EVs

Diluent solutions of OptiPrep density gradient medium (Sigma-Aldrich, D1556) were prepared and loaded bottom to top (5 to 50%) in the 13.2-ml ultraclear centrifugation tube (Beckman, 344058). EVs were loaded onto the top of OptiPrep density gradient tube and then centrifuged using SW41 Ti rotor at 200,000*g* for 18 hours. Ten fractions (1 ml each) were collected from the bottom of tube. All fractions were diluted with filtered PBS and centrifuged again to pellet EVs using SW41 Ti rotor at 100,000*g* for 2 hours. EV pellets were resuspended in filtered PBS.

### Western blotting and IP

Cell lysates were prepared in M-PER buffer (Thermo Fisher Scientific, 78501) supplemented with protease inhibitors (Roche). For Western blotting, cell lysates and EVs were resuspended in NuPAGE LDS sample buffer (Novex, NP0008) with NuPAGE sample reducing agent (Novex, NP0009). IP was carried out using the FLAG immunoprecipitation kit (Sigma-Aldrich). Cell lysates were incubated in anti-FLAG agarose affinity gel at 4°C overnight. Immunoprecipitated samples were washed three times with washing buffer, eluted, and subjected to Western blotting. All Western blotting samples were loaded on a 4 to 12% NuPAGE gel or 12% NuPAGE gel and transferred onto 0.22-μm polyvinylidene difluoride membrane (Bio-Rad, 1620177). Primary antibodies include anti-FLAG antibody (Sigma-Aldrich, F1804; 1:2000 dilution), anti-vinculin antibody (Abcam, ab129002; 1:2000 dilution), anti-CD9 antibody (Cell Signaling Technology, 1317S; 1:1000 dilution), anti-Scamp3 antibody (GeneTex, GTX102216; 1:2000 dilution), and anti-ARRDC1 antibody (in-house; 1:3000 dilution). Secondary antibodies include horseradish peroxidase (HRP)–conjugated anti-rabbit (Cell Signaling Technology, 7074S; 1:2000 dilution) and HRP-conjugated anti-mouse (Cell Signaling Technology, 7076S; 1:2000 dilution).

### Immunogold labeling and electron microscopy of EVs

EVs prepared via OptiPrep density gradient purification were absorbed for 1 min to a carbon-coated grid (Electron Microscopy Sciences, CF400-CU) that had been made hydrophilic by a 20-s exposure to a glow discharge. For immunogold labeling, 1% bovine serum album in PBS was used for blocking for 10 min. Diluted anti-M2 antibody (GeneTex, GTX 125951) in 1% bovine serum album in PBS was used to incubate with the grid for 30 min. The grid was then washed three times by PBS for 10 min, incubated with protein A–10-nm colloidal Gold Labeled (Sigma-Aldrich) in 1% bovine serum album in PBS for 20 min, and washed by PBS for 5 min and water for 10 min consecutively. Excess liquid was removed with a filter paper. The grid was stained with 0.75% uranyl formate for 30 s and examined using a JEOL 1200EX transmission electron microscope.

### LC-MS/MS and data analysis

The protein sequence analysis of EVs was carried out by the Taplin Biological Mass Spectrometry Facility at the Harvard Medical School. All EVs samples were stained with Coomassie blue stain after gel-running. Each lane of Coomassie blue–stained SDS-PAGE was cut into approximately 1 mm for three pieces, and all samples were subjected to a modified in-gel trypsin digestion procedure. Briefly, the gel pieces were dehydrated with acetonitrile for 10 min and were completely dried in SpeedVac. Fifty millimolars of ammonium bicarbonate solution containing modified sequencing-grade trypsin (12.5 ng/μl) (Promega, Madison, WI) was used for rehydration of the gel pieces. Digestion was proceeded in 37°C for 16 hours. Peptides were reconstituted in 5 to 10 μl of high-performance LC solvent A (2.5% acetonitrile and 0.1% formic acid). Each sample was loaded onto a homemade reversed-phase analytical column (100 μm by 30 cm) packed with 2.6 μm of C18 spherical silica beads after preequilibration. A flow rate of 300 nl/min and 60 min of the gradient from 5% solvent B (95% acetonitrile and 0.1% formic acid) to 38% solvent B was applied for the separation of peptides. Eluted peptides were subjected to electrospray ionization and were entered into an LTQ Orbitrap Velos Pro ion-trap mass spectrometer (Thermo Fisher Scientific, Waltham, MA). MS scan range was from 350 to 1250 mass/charge ratio (*m*/*z*) with the resolution of 60,000. For MS/MS, the scan range was set to 2000 *m*/*z*. A tandem mass spectrum of specific fragment ions for each peptide was produced by the fragment of peptides. Identification of peptide sequences was performed by matching UniProt Human database (release 2017_06_20; 80,010 entries included a reversed version of all the sequences) with the acquired fragmentation pattern by the software program Sequest (Thermo Fisher Scientific, Waltham, MA). The maximum of missed cleavages is 2. The mass tolerance values for precursor and fragment ions were 50 parts per million and 1 Da, respectively. Both maximum peptide and protein false discovery rates were limited to 1%. Protein quantification was performed by intensity, which was based on at least two unique peptides to quantify the different protein profiling in the EVs. Only those proteins that can be detected in all three biological replicates were retained. Quantile normalization was performed to ensure that each sample had the same distribution; the twofold change and *P* < 0.05 cutoff were set for the screening of differentially expressed proteins. The Database for Annotation, Visualization, and Integrated Discovery (DAVID) (https://david.ncifcrf.gov/) was used for pathway analysis of 124 M2-WAEV proteins, which has not been detected in previous exosome database.

### Mice immunization and sera collection

All animal experiments were approved by the Harvard Medical Area Institutional Animal Care and Use Committee under the protocol #IS506-6. Healthy male CD-1 IGS mice (strain code: 022) and BALB/c (strain code: 028) mice were purchased from Charles River Laboratories (Wilmington, MA) and housed in microisolator full sterile technique cages in a barrier animal facility. For EV immunization, mice were administrated with 5 × 10^9^ EVs intraperitoneally (200 μl per mouse) one time or three times with 2-week intervals. Serum was obtained by retro-orbital bleeding after anesthesia by ketamine (90 mg/kg) and xylazine (10 mg/kg) solution 3 days after final immunization.

### Mouse model of influenza infection

Mice were anesthetized with ketamine (72 mg/kg) and xylazine (9.6 mg/kg) by intramuscular injection and infected intranasally with 25 μl of influenza A virus (a murine-adapted strain of H1N1, A/Puerto Rico/8/1934; ViraSource, Durham, NC) quantified as plaque-forming units. After influenza infection, mice were monitored daily for 14 days to measure body weight and observe symptom severity for evidence of influenza-related clinical disease including weight loss, morbidity, and mortality.

### Enzyme-linked immunosorbent assays

Influenza A or HIV-specific IgG titers in sera from immunized mice were measured by ELISAs. Influenza A virus (a murine-adapted strain of H1N1, A/Puerto Rico/8/1934; ViraSource, Durham, NC) and HIV pseudo-virus (Cap45) were inactivated in 70°C for 30 min. Heat-inactivated virus (500 ng) were used to coat 96-well plate with 1% bovine serum album in PBS overnight. Diluted sera were used to incubate with the virus-coated plates. HRP-conjugated anti-mouse IgG antibody (Cell Signaling Technology, 7076S; 1:4000 dilution), anti-mouse IgG1 antibody (Thermo Fisher Scientific, PA1-74421; 1:4000 dilution), and anti-mouse IgG2a antibody (Thermo Fisher Scientific, M32207; 1:4000 dilution) were loaded as a secondary antibody. Substrate reagent pack (R&D Systems, DY999) and stop solution (R&D Systems, DY994) were used to detect antigen-specific antibody titer signal.

### HIV neutralization assay

The assay was done according to a published method (*48*). Briefly, HIV pseudo-virus (YU2) was mixed with mouse sera or the control purified recombinant 2F5 antibody. Virus-serum mixture were incubated at 37°C for 90 min and added to TZM-bl cells. One day after infection, cells were washed with PBS, and fresh medium was added to the cells. Three days after infection, the supernatant was removed, and the cells were washed with PBS and then lysed with a luciferase assay kit (Bright-Glo, E2620). The plates were measured for luciferase activity using a luminometer.

### Statistical analyses

All statistics analyses were performed by GraphPad Prism software. Unpaired *t* test was used in comparison of two groups for NTA results analysis, and two-way analysis of variance (ANOVA) test was used in comparison among multiple groups for antibody titer, body weight, and morbidity score. All survival rates were analyzed via Gehan-Breslow-Wilcoxon test. *P* < 0.05 was considered significant. *P < 0.05, **P < 0.01, and ***P < 0.001. All data were reported as the means ± SD.

## Notes

View/request a protocol for this paper from *Bio-protocol*.
