# Supplementary material for: Displaying and delivering viral membrane antigens via WW domain–activated extracellular vesicles
Source: Sci Adv. 2023 Jan 27;9(4):eade2708. doi: 10.1126/sciadv.ade2708 (PMC9882979; doi:10.1126/sciadv.ade2708)
Supplement: Supplementary file 1 — Figs. S1 to S8 [file sciadv.ade2708_sm.pdf]

Science Advances
AAAS

# Supplementary Materials for

## Displaying and delivering viral membrane antigens via WW domain–activated extracellular vesicles

Sengjin Choi *et al.*

Corresponding author: Quan Lu, qlu@hsph.harvard.edu

*Sci. Adv.* **9**, eade2708 (2023)
DOI: 10.1126/sciadv.ade2708

**The PDF file includes:**

Figs. S1 to S8
Legends for tables S1 to S3

**Other Supplementary Material for this manuscript includes the following:**

Tables S1 to S3

**Supplementary Figure S1.**

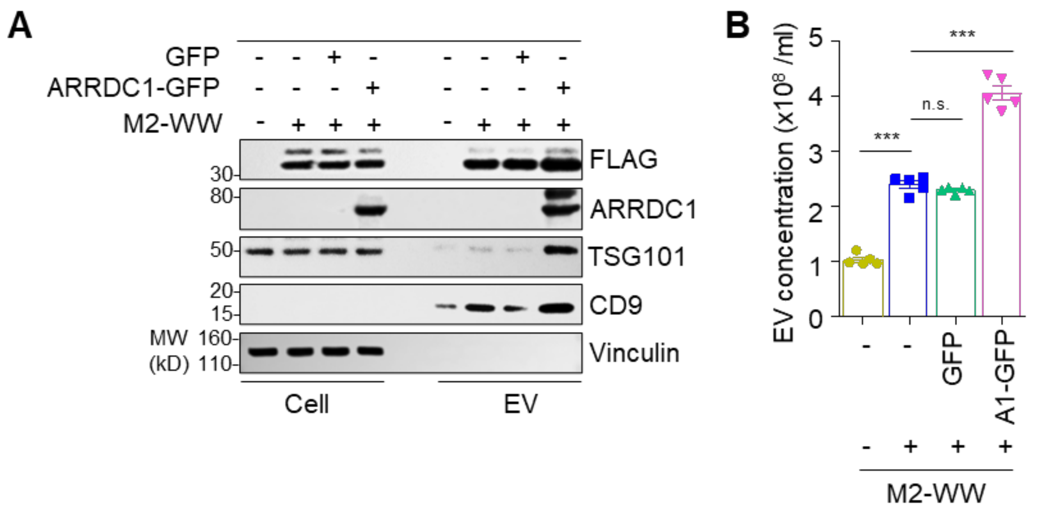


**Fig. S1. Effect of co-expression of ARRDC1 in M2-WAEV budding from HEK293T cells**. **(A)** Western blotting result showing budding of M2-WAEV into EVs and **(B)** NanoSight particle analysis of EVs from HEK293T cells transfected with vector control, M2-WW, M2-WW with GFP, and M2-WW with ARRDC1-GFP. ***p<0.001, n.s., not significant.

**Supplementary Figure S2.**

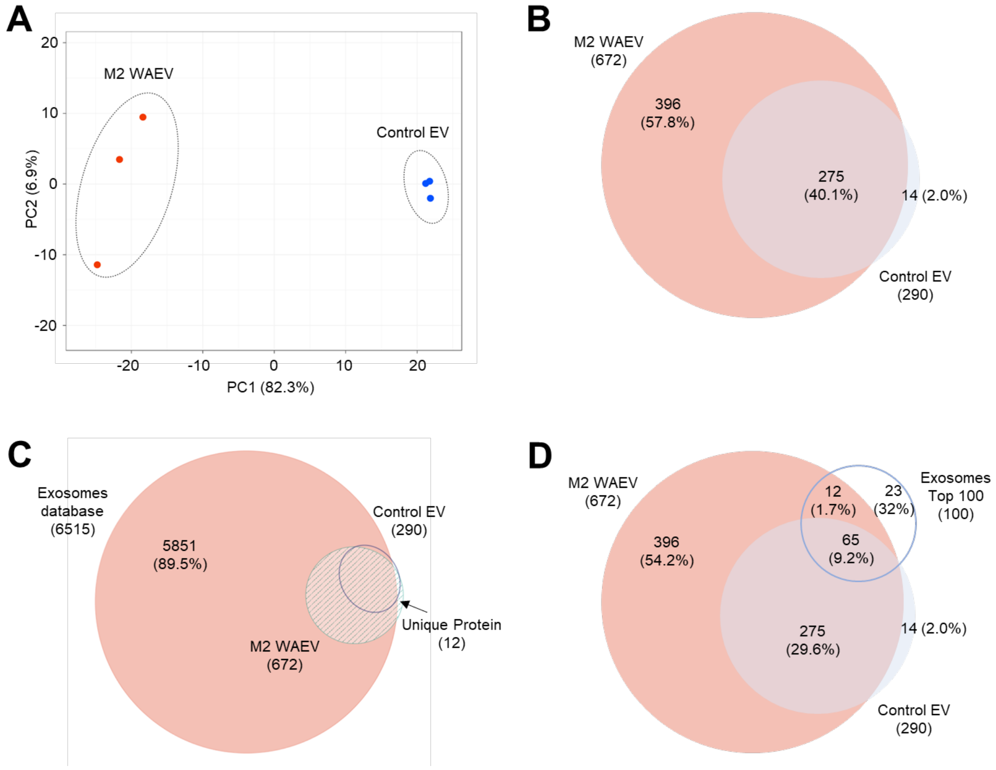


**Fig. S2. Analysis of M2-WAEV proteomic datasets. (A)** Principal component analysis (PCA) of Control EV and M2-WAEV proteomics sample (three biological repeats). **(B)** Overlap of control EV and M2-WAEV proteins. **(C)** Overlap of control EV proteins, M2-WAEV proteins, and exosomal proteins (data acquired from ExoCarta). **(D)** Overlap of control EV proteins, M2-WAEV proteins, and top 100 exosomal proteins (data acquired from ExoCarta).

**Supplementary Figure S3.**

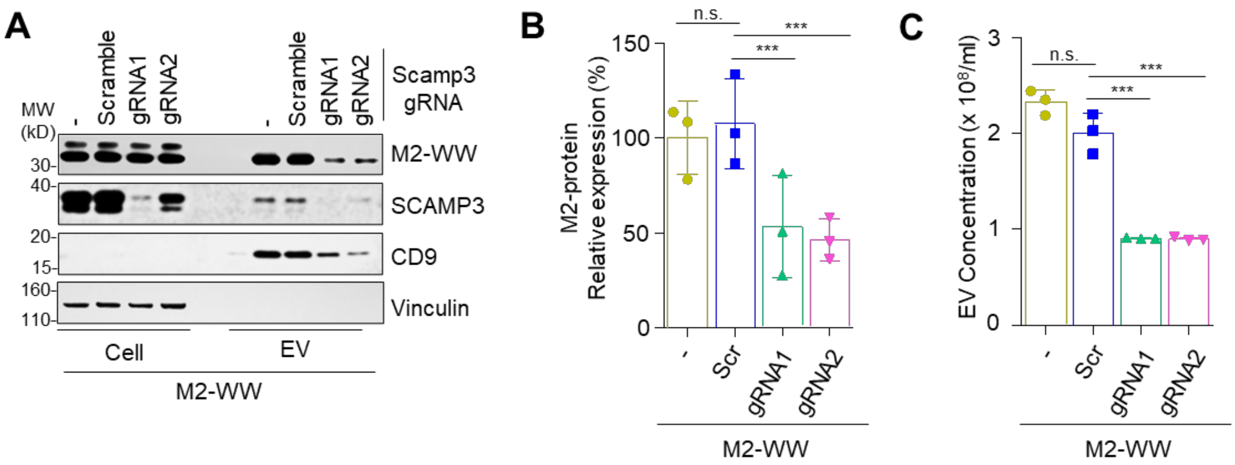


**Fig. S3. Effect of CRISPR-Cas9 knockdown of *SCAMP3* on M2-WAEV budding.** HEK293T cells transduced with lentiviruses containing scrambled guide RNA or SCAMP3-targeting guide RNAs were transfected with M2-WW. (**A**) EVs were isolated from the cells and subjected to Western blotting. **(B)** Analysis of Western blotting bands via image quantification. (**C**) NanoSight particle analysis. Data were obtained from triplicates for each condition. ***p<0.001. n.s., not significant.

**Supplementary Figure S4.**

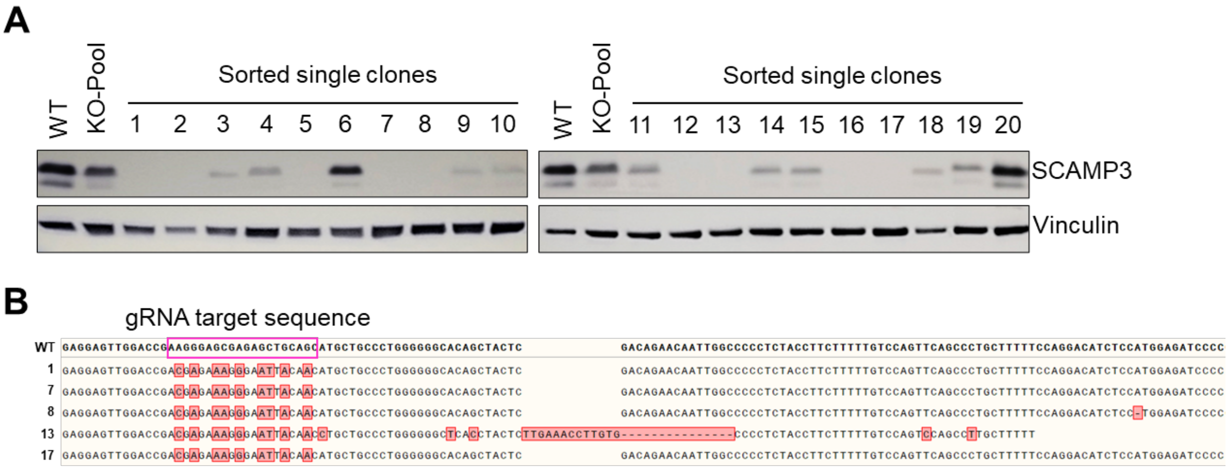


**Fig. S4. Isolation and sequencing of single-sorted *SCAMP3*-Knockout cell clones. (A)** Western blotting of single-sorted cell clones. **(B)** sequence alignment between WT cells and multiple *SCAMP3* Knockout clones.

**Supplementary Figure S5.**

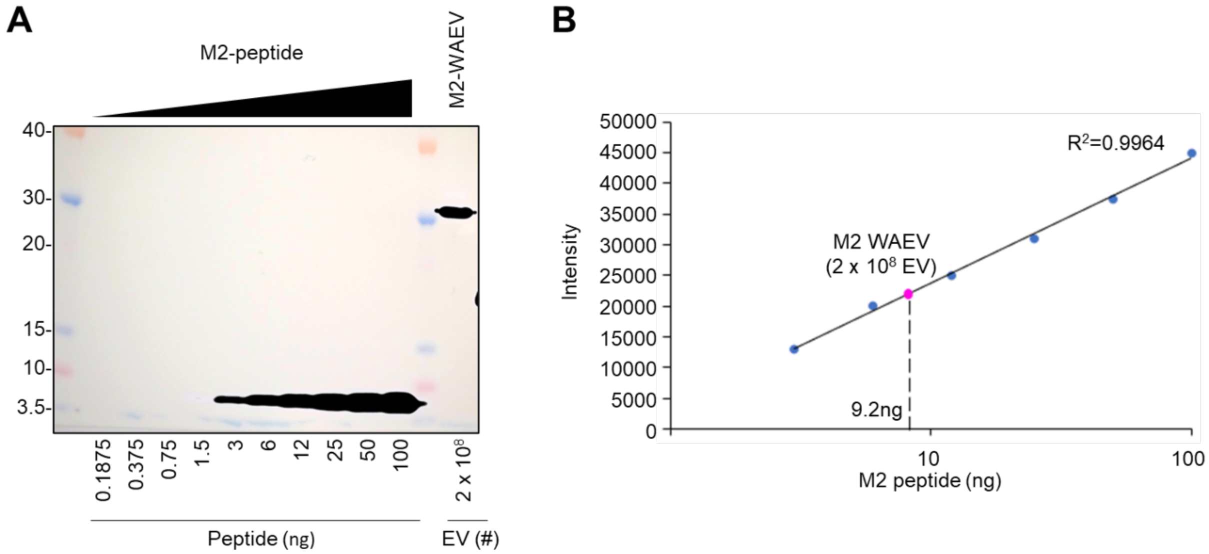


**Fig. S5. Measurement of amount of M2 antigen on M2 WAEV. (A)** Western blotting of serial diluted synthesized M2 peptide and M2-WAEV. Anti-M2 antibody was used for the Western blotting. **(B)** Analysis of M2 WAEV band intensity using standard curve of M2 peptide. Protein bands in the Western blotting were quantified by ImageJ (Analyze/Gels function) and analyzed in Excel: M2 on 2 x $10^8$ WAEVs has a mass of 9.2 ng. Our subsequent calculation indicated that each WAEV vesicle contains an average of ~ 900 M2-WW protein molecules.

**Supplementary Figure S6.**

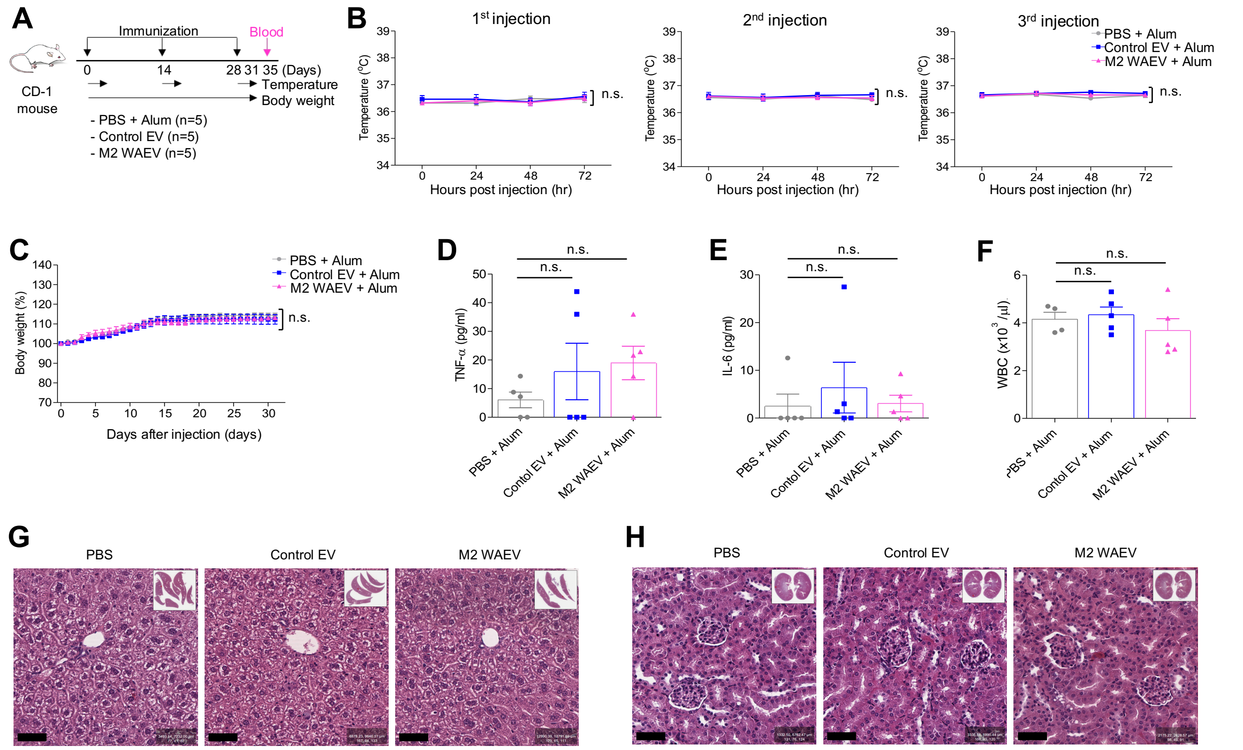


**Fig. S6. Toxicity test of M2-WAEV immunization in mice. (A)** CD-1 mice (Charles River) were immunized via intraperitoneal (i.p.) injection three times over a 4-week period with one of the following: PBS with Alum, control EVs with Alum, and M2-WAEVs with Alum. Sera were collected from mice one week after the final immunization. Schematic of immunization protocol. **(B)** Measurement of body weight measurements of immunized mice 24,48, and 72 hours after each injection. **(C)** Daily body weight measurements of immunized mice from day 0 to day 31. **(D)** The level of TNF-α and **(E)** IL-6 in serum of immunized mice one week after the final immunization. n.s., not significant. **(F)** Number of White blood cells (WBC) in blood from immunized mice one week after the final immunization. n.s., not significant. **(G)** Tissue histology image (H&E staining) of liver and **(H)** kidney extracted from the immunized mice one week after the final immunization.

**Supplementary Figure S7.**

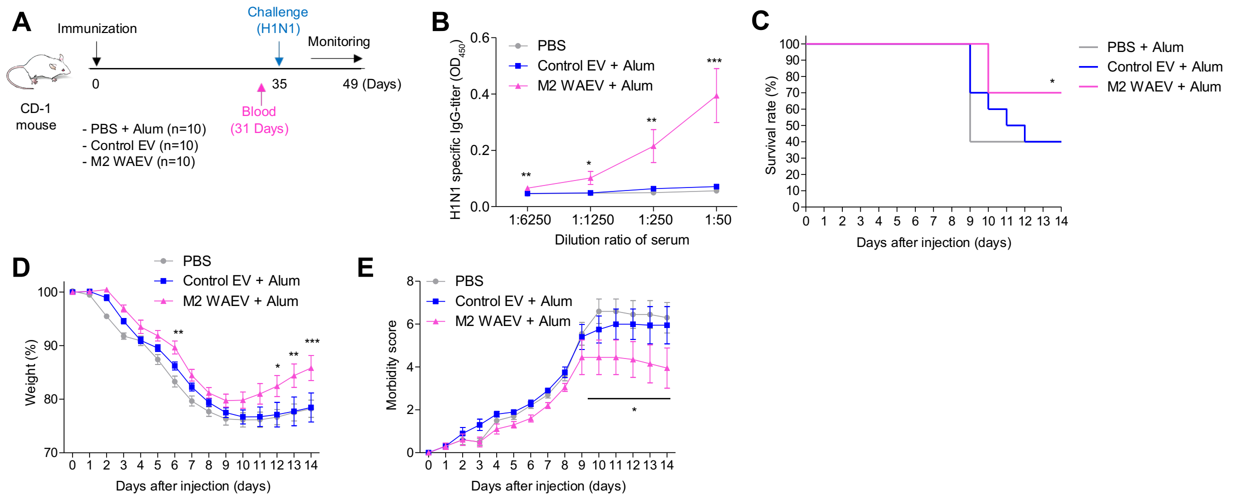


**Fig. S7. Effect of single-shot M2-WAEV immunization on flu viral lethality (A)** Schematic of single-shot immunization protocol. CD-1 mice (Charles River) were immunized via intraperitoneal (i.p.) injection with one of the following: PBS, control EVs, or M2-WAEVs (all with Alum). Sera were collected from mice 31 days after immunization and were used to measure H1N1-virus binding IgG. Five weeks after the immunization, all mice were subjected to H1N1 influenza viral infection (strain A/Puerto Rico/8/1934/H1N1 at 800 PFU; given intranasally) **(B)** Levels of H1N1-reactive IgG in serum from mice on days 31. **(C)** Survival rate of immunized mice after influenza virus infection. **(D)** Weight measurement in immunized mice after influenza virus infection. **(E)** Morbidity score in immunized mice after influenza virus infection. *p<0.05, **P<0.01, ***p<0.001, n.s., not significant.

**Supplementary Figure S8.**

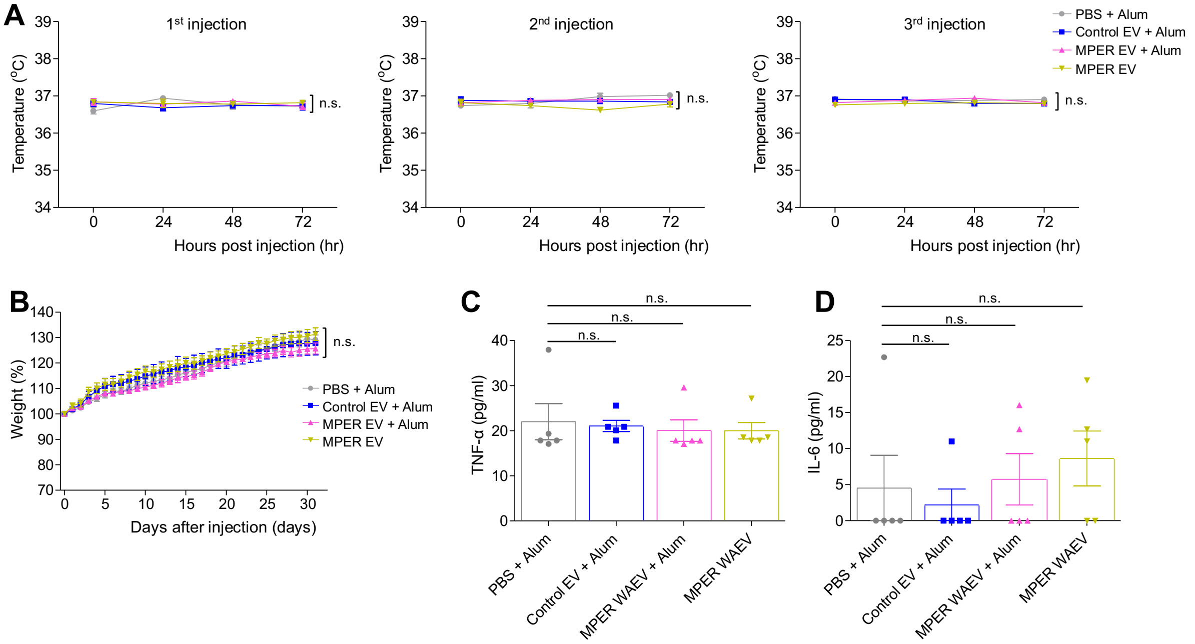


**Fig. S8**. **Toxicity test of MPER-WAEV immunization in mice.** BALB/c mice were immunized via intraperitoneal (i.p.) injection three times over a 4-week period with one of the following: PBS with Alum, control EVs with Alum, MPER-WAEVs with Alum, or MPER-WAEVs without Alum. Sera were collected from mice one week after final immunization. **(A)** Measurement of body temperature of immunized mice after 24,48, and 72 hours post each injection. **(B)** Daily weight measurement in immunized mice on day 0 – 31. **(C)** The level of TNF-α and **(D)** IL-6 in serum from immunized mice one week after final immunization. n.s., not significant.

**Supplementary Table S1. 362 specific proteins in M2 WAEV.** Comparison of the mass spectrometry results from M2-WAEVs and control EVs.

**Supplementary Table S2. 164 enriched proteins (>2 fold) in M2 WAEV.** Comparison of the mass spectrometry results from M2-WAEVs and control EVs.

**Supplementary Table S3. 12 unique proteins in M2 WAEV.** Comparison of WAEV proteomic dataset with known exosomal protein database (6515 proteins)
